# Combination therapy of cisplatin and resveratrol to induce cellular aging in gastric cancer cells: Focusing on oxidative stress, and cell cycle arrest

**DOI:** 10.3389/fphar.2022.1068863

**Published:** 2023-01-04

**Authors:** Mahban Rahimifard, Maryam Baeeri, Taraneh Mousavi, Asaad Azarnezhad, Hamed Haghi-Aminjan, Mohammad Abdollahi

**Affiliations:** ^1^ Toxicology and Diseases Group (TDG), Pharmaceutical Sciences Research Center (PSRC), Tehran University of Medical Sciences (TUMS), Tehran, Iran; ^2^ Liver and Digestive Research Center, Research Institute for Health Development, Kurdistan University of Medical Sciences, Sanandaj, Iran; ^3^ Pharmaceutical Sciences Research Center, Ardabil University of Medical Sciences, Ardabil, Iran

**Keywords:** aging, chemotherapy, cisplatin, gastric cancer, polyphenol, resveratrol

## Abstract

**Background:** As a medical dilemma, gastric cancer will have 7.3 million new cases in 2040. Despite the disease’s high economic and global burden, conventional chemotherapy regimens containing cisplatin have insufficient effectiveness and act non-specifically, leading to several adverse drug reactions To address these issues, the biological efficacy of the cisplatin-resveratrol combination was tested.

**Methods:** To find IC50, gastric adenocarcinoma cells (AGS) were exposed to different concentrations of resveratrol and cisplatin. Anti-cancer and anti-metastatic effects of 100 M resveratrol with concentrations of cisplatin (25, 50, and 100 g/ml) were studied by assessing ß-galactosidase and telomerase activities, senescence and migration gene expression, reactive oxygen species (ROS) level, and cell cycle arrest.

**Results:** Co-administration of cisplatin and resveratrol increased ß-galactosidase activity, ROS level as a key marker of oxidative stress, p53, p38, p16, p21, and MMP-2 gene expression, and induced G0/G1 cell cycle arrest. Additionally, telomerase activity, pro-inflammatory gene expression, and cell invasion were suppressed. The best results were achieved with 100 g/ml cisplatin co-administered with resveratrol.

**Conclusion:** The current study proved the synergistic effect of the cisplatin-resveratrol combination on suppressing metastasis and inducing apoptosis and cell senescence through targeting P38/P53 and P16/P21 pathways. Such promising results warrant translation to animal models and the clinic. This may lead to cost-effective, available, and accessible treatment regimens with targeted action and the fewest ADRs.

## 1 Introduction

Cancer was once considered as an incurable disease, but today, thanks to advances in science, there is a great hope for treatment of individuals with the early stage diagnosed disease. According to the World Health Organization’s 2022 report, cancer would account for over 10 million deaths in 2020, making it the leading cause of death globally. Amongst, lung, breast, colon, and rectum, stomach, prostate, and skin cancers have the greatest number of new cases in 2020. Almost similarly, the most common causes of death were due to lung, colon and rectum, liver, stomach, and breast cancers (WHO, 2022a). Looking in detail, in 2022, the estimated number of worldwide incidence and deaths of gastric cancer is 1,089,103 and 768,793, respectively (WHO, 2022b). More to the point, the estimated number of new gastric cancer cases will undergo a considerable rise, reaching 1.2 (males) and 6.1 (females) million cases in 2040. Likewise, gastric cancer is expected to cause 859, 600 (males) and 450, 900 (females) deaths in 2040 (WHO, 2022c). Therefore, despite all recent advances in the field of oncology, defeating this medical dilemma requires a substantial effort; particularly in case of gastric cancer which is usually poor prognosis and detected at an advanced stage (Ajani et al., 2022).

Based on the staging of gastric cancer, different treatment approaches from surgery to palliative management and targeted therapies may be indicated. FLOT (docetaxel, oxaliplatin, leucovorin and fluorouracil), ECX (epirubicin, cisplatin, and capecitabine), ECF (epirubicin, cisplatin, and fluorouracil), EOX (epirubicin, oxaliplatin, and capecitabine), CAPOX (oxaliplatin and capecitabine), and FOLFOX (oxaliplatin, leucovorin, fluorouracil) regimens are among the preferred options based on the disease condition (Advisor CT, 2022; Ajani et al., 2022). Considering targeted therapies, nivolumab, pembrolizumab, and trastuzumab are recommended by National Comprehensive Cancer Network (NCCN) 2022 guideline for locally advanced or metastatic gastric cancer (Ajani et al., 2022). Despite the advancement of chemotherapy over the recent years, some of the currently used non-targeted medications have poor performance in achieving tumor location. Moreover, due to non-specific distribution, they are associated with several adverse reactions, reducing patients' compliance. For instance, common cisplatin-related adverse reactions are neurotoxicity (esp, in form of peripheral neuropathy), nausea and vomiting, nephrotoxicity (e.g., acute renal failure and chronic renal insufficiency), anemia, leukopenia, thrombocytopenia, elevated hepatic enzymes, and ototoxicity (e.g., tinnitus and hearing loss) (USFDA, 2019).

Since the ultimate goals in the treatment of various cancers are palliation, improving survival and quality of life, the best therapeutic regimen should specifically target tumor site and reduced adverse drug reactions to the best possible level. To this end, a great body of evidence have suggested mitochondrial targeting as a promising approach in the future management of gastric cancer (Tanprasert et al., 2022). Chemotherapeutic agents also exert their effects through inducing mitochondrial apoptosis. As a polyphenol flavonoid, 3, 4, 5-trihydroxy-trans-stilbene (resveratrol) demonstrated protective effects against incidence and progression of gastric cancer (Wang et al., 2020; Ashrafizadeh et al., 2021a). This is related to its *Helicobacter pylori* (*H. pylori*) bactericidal feature along with affecting oxidative stress, cell survival, apoptosis, and migration of cancerous cells (Wang et al., 2020; Ashrafizadeh et al., 2021a). Accordingly, co-administration of resveratrol with any of the indicated medications seems promising and worth further investigation in the preclinical and clinical levels.

Considering the positive effects of resveratrol (HaghiAminjan et al., 2019; Amini et al., 2021) as well as non-specificity, high cost and several side effects of chemotherapy agents (Samadi et al., 2021), this work carefully focuses on the potential of resveratrol to cause cytotoxicity and cellular aging as well as preventing cell migration and gastric tumor cells inflammation. Besides, the results will take a step towards gastric cancer management through targeting intracellular pathways and controlling genes expression involved in tumorigenesis as well as reducing metastasis.

## 2 Materials and methods

### 2.1 Chemicals

Cisplatin was obtained from Mylan (France). Resveratrol, 2′,7′-dichlorodihydrofluorescein diacetate (DCFH-DA), and 3-(4,5 dimethylthiazol-2-yl)-2,5-diphenyltetrazolium bromide (MTT) from Sigma-Aldrich (Munich, Germany). Primers was obtained from eurofins genomics (Germany). The β-galactosidase kit was purchased from Cusabio Biotech (China). TeloTAGGG Telomerase PCR kit was from (Roche, Germany). Total RNA Prep kit, BioFact™ 5 × RT Pre-Mix and BioFACT™ 2× Real-Time PCR Master Mix were supplied from BioFact (Seoul, Korea).

### 2.2 Ethics approval

All experiments were conducted according to the Ardabil University Medical Sciences (ARUMS) ethical approval number IR. ARUMS.REC.1398.653.

### 2.3 Cell culture

The AGS cell line (a human gastric adenocarcinoma cell-line) and HEK293 cell line (a human embryonic kidney) were obtained from Iran’s Pasteur Institute (Tehran, Iran). The cells were grown in DMEM with high glucose, 10% FBS, 100 U/mL penicillin, and 100 g/ml streptomycin sulfate (growth medium). A humidified environment with a CO_2_ content of 5% was used to incubate the cells at 37°C for 24 h.

### 2.4 Evaluation of the cytotoxic effects of cisplatin and resveratrol on AGS cell viability by MTT method

In this study, AGS and HEK293 cells were cultured and exposed to different concentrations of cisplatin and resveratrol. To determine cisplatin half-maximal inhibitory concentration (IC_50_), AGS cells were cultured and exposed to various concentrations of cisplatin and resveratrol. Then, cells were planted in a 96-house plate with 1*10^5^ in each well. After 24 h, treatment of cells with different concentrations of cisplatin (0.01, 0.1, 1, 10, 100 μg/ml) and resveratrol (0.01, 0.1, 1, 100, 10, 100, 1,000 μM) was performed. Forty-8 hours post treatment, toxicity of resveratrol and cisplatin were assessed. Later, 50 μL of MTT solution (5 mg/ml) was added, and the mixture was incubated for 3 h at 37°C in a humidified environment with 5% CO_2_. Finally, 150 μL of DMSO solution was added, and the absorbance was measured using an ELISA reader at 570 nm (BioTek^®^, Synergy™ HT, USA).

### 2.5 Evaluation of combination index (CI) of cisplatin and resveratrol

The combination index (CI) of Cisplatin and Resveratrol was evaluated using Compusyn Software to analyze the anticancer effect of them. After analyzing MTT assay data, relative cell viability (% of control) was calculated. Then, using Chou-Talalay method, CI was evaluated.

### 2.6 Study design and treatment groups

Based on the results of the MTT assay, the amount of Cis IC_50_ was calculated to be 100 μg/ml. After determining the IC_50_ of cisplatin and the effective concentration of resveratrol, the studied cells were divided into four groups:

1) Control group: AGS cells under normal cell culture conditions.
2) Groups 2–4 wherein AGS cells were exposed to IC_50_, ½IC_50_ and ¼IC_50_ equivalent to 100, 50 and 25 μg/ml of cisplatin and 100 μM of resveratrol, respectively.

Oxidative stress parameters, inflammatory markers, ß-galactosidase and telomerase activities, cell cycle arrest, and mRNA expression of genes associated with cellular aging, inflammation, and migration were evaluated thereafter.

### 2.7 Assessment of β-galactosidase activity associated with cellular aging

This experiment was carried out according to the manufacturer’s instructions using a human-specific ß-galactosidase kit. The optical density of each well was read at 450 nm for 5 min.

### 2.8 Protein assay

The Bradford protein assay was carried out using a spectrophotometer at 595 nm in accordance with the methodology that has been reported previously (Bradford, 1976).

### 2.9 Measurement of free radicals by reactive oxygen species (ROS) assay

The content of ROS assayed according to ROS kit protocol. Finally, it is determined in 520 nm by ELISA fluorimeter and calculated based on standard curve. Finally, the oxidation of DCFH to DCF at wavelength (Ex: 485/20/Em: 528/20) was measured every 5 min for 1 h by ELISA fluorimeter (BioTek, SynergyTM HT, United States) (Bagheri et al., 2018). H_2_O_2_ was used as a positive control at a concentration of 100 μM.

### 2.10 Evaluation of *p53*, *p38, p16*, *p21*, *TNF-*α, *IL-1*β, *IL-6* and *MMP-2* gene expression by real-time polymerase chain reaction (PCR)

In order to evaluate the level of gene expression for *p53, p38, p16, p21, TNF-*α, *IL-1, IL-6*, and *MMP-2* using particular primers and real-time PCR. In the first step, mRNA was extracted from the cells using the appropriate kit technique. Using a Nano-Drop UV-vis spectrophotometer, we were able to determine the concentration of RNA (Thermo Fisher Scientific, CA). cDNA was then manufactured using an iScript cDNA synthesis kit after that. Primers that are specific for the genes described were developed by NCBI primer three and obtained from Eurofins genomics (Eurofins genomics, Germany). In conclusion, a real-time PCR reaction was performed with a SYBR green master mix utilizing a Light Cycler 96. (Roche Applied Sciences, USA). For analysis, after obtain cycle number (Ct) of each reaction were normalized by ACTB mRNA and the relative gene expression level was represented as 2^−ΔΔCt^. The following primers were used in this study:

ACTB F: AAA​ACT​GGA​ACG​GTG​AAG​GT.

ACTB R: AAC​AAC​GCA​TCT​CAT​ATT​TGG​AA

p53 F: CGT​GTG​GAG​TAT​TTG​GAT​GAC

p53 R: TTG​TAG​TGG​ATG​GTG​GTA​CAG​TC

p38 F: GGT​TAC​GTG​TGG​CAG​TGA​AG

p38 R: AGA​TCT​GCC​CCC​ATG​AGA​TG

p21 F: GGC​ACC​CTA​GTT​CTA​CCT​CA

p21 R: CTC​CTT​GTT​CCG​CTG​CTA​AT

p16 F: CAG​TCA​CCG​AAG​GTC​CTA​CA

p16 R: TTT​ACG​GTA​GTG​GGG​GAA​GG.

IL-6 F: TGC​CTG​GTG​AAA​ATC​ATC​ACT​G.

IL-6 R: CAG​CTC​TGG​CTT​GTT​CCT​CA.

IL-1β F: GAC​CAC​CAC​TAC​AGC​AAG​GG.

IL-1β R: GTG​CAT​CGT​GCA​CAT​AAG​CC.

TNF-α F: GCT​GCA​CTT​TGG​AGT​GAT​CG.

TNF-α R: CTT​GTC​ACT​CGG​GGT​TCG​AG.

MMP-2 F: ATG​GCG​ATG​GAT​ACC​CCT​TT.

MMP-2 R: TCC​CAT​ACT​TCA​CAC​GGA​CC.

### 2.11 Investigation of cell cycle by flow cytometry analysis

Propidium iodide (PI) is a non-specific DNA probe that forms a fluorescent complex in DNA. PI is excited by UV and emits red fluorescence. The intensity of the fluorescence indicates the DNA content. Flow cytometry is a suitable tool for measuring the state of cell cycle by evaluating the content of cellular DNA. After treated cells in each group, according to the kit protocol, cells were fixed in cold 70% ethanol and then stained with 50 μg/ml PI containing 20 μg/ml RNase. Finally, they were assayed with flow cytometry (Mindray, China) and analyzed by FlowJo software (Rahimifard et al., 2020).

### 2.12 Evaluation of cell migration by scratch method and microscopic examination

The cells were distributed at a density of 10^4^ in each well in a 96-well microplate and incubated at 37 °C for 24 h to adhere to the bottom of the container. Then, a scratch was made in the middle of each well with a sterile yellow sampler. After washing the cells with phosphate buffer, agents were added. Following 48 h, the migration of cells to the scratched area was examined under a microscope and photographed. Finally, the rate of cell migration was quantitatively analyzed using ImageJ software (Sarkhail et al., 2020).

### 2.13 Telomerase activity

Following treatment of cells with resveratrol and different concentrations of cisplatin and incubation for 24 h, cellular extract was provided using lysate reagent. Then, 1–3 μL of cell lysate was added to biotin primers and nucleotides. The cellular enzyme telomerase, added telomeric repeats to the ends of biotin primers. In the next step, the telomeric products were subjected to PCR reaction and finally the PCR products were evaluated by the ELISA method. In this case, the amount of telomerase activity is proportional to the intensity of the dye created, and based on the formula in the kit instructions, the relative activity of telomerase enzyme was calculated (Baeeri et al., 2018).

### 2.14 Statistical analysis

Stats-Direct version 3.0.183 software was used for statistical calculations of data. The statistical test used is one-way analysis of variance (ANOVA) with multiple comparisons (*post hoc* Tukey’s test). The results of the experiments were presented as Mean ± SD. Results with a *p*-value less than 0.05 were considered as statistically significant.

## 3 Results

### 3.1 Toxicity effect of different concentrations of resveratrol and cisplatin on the viability of AGS cells

**FIGURE 1:**
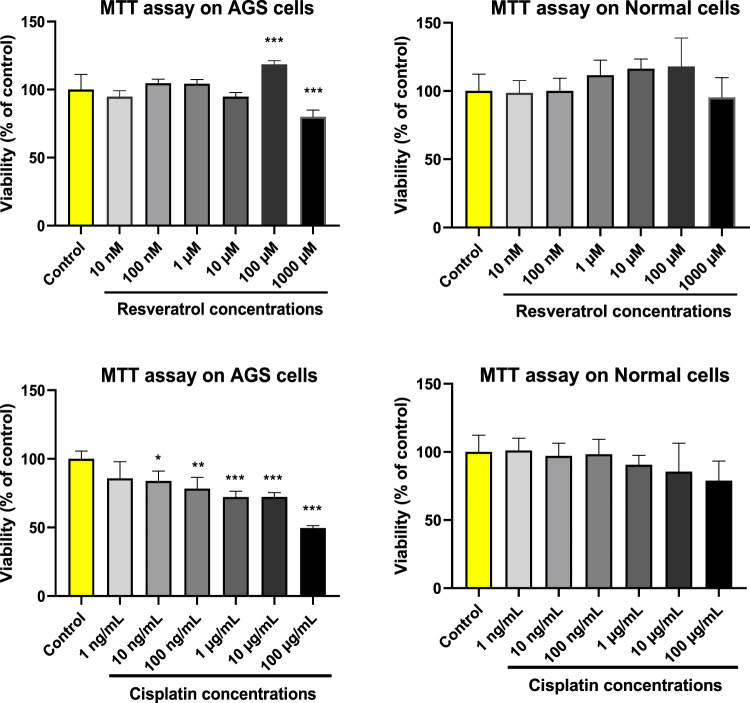
Cytotoxicity effects of different concentrations of resveratrol (RES) and cisplatin (CIS) on the viability of adenocarcinoma gastric cells (AGS) and normal HEK293 cell line. The results are expressed as mean ± SD performed on *n* = 6, with two independent replicates. *** (*p* < 0.001), ** (*p* < 0.01), and * (*p* < 0.05) show a significant difference compared to the control group.

The MTT assay was performed to investigate the cells exposed to different concentrations of resveratrol and cisplatin. As shown in Figure 1, changes in viability of the normal cells were not significantly. About the AGS cells treated with resveratrol, only 1,000 μM resveratrol was toxic and the others were safe. Among those concentration, 100 μM resveratrol made a significant increment in viability of the AGS cells (*p* < 0.001). So, this concentration was considered as effective dose in this study. In the other hand, tested concentrations of cisplatin decreased viability of the AGS cells dose dependently. According to the obtained results, the IC_50_ of cisplatin was estimated at 100 μg/ml by probit regression (Version 11.5, SPSS Inc, Chicago, IL). In the following, cells were exposed to IC_50_, ½ IC_50_ and ¼ IC_50_ (100, 50, and 25 μg/ml) of cisplatin.

### 3.2 Combination index (CI) of resveratrol and cisplatin

According to the MTT assay data, the possible synergistic effect of Resveratrol and Cisplatin was calculated by the combination index (CI) assays using Compusyn software. At Fa 0.5, CI value was evaluated as 0.55, which indicates synergistic effects of these two compounds.

### 3.3 Effect of resveratrol and different concentrations of cisplatin on AGS ß-galactosidase activity

**FIGURE 2:**
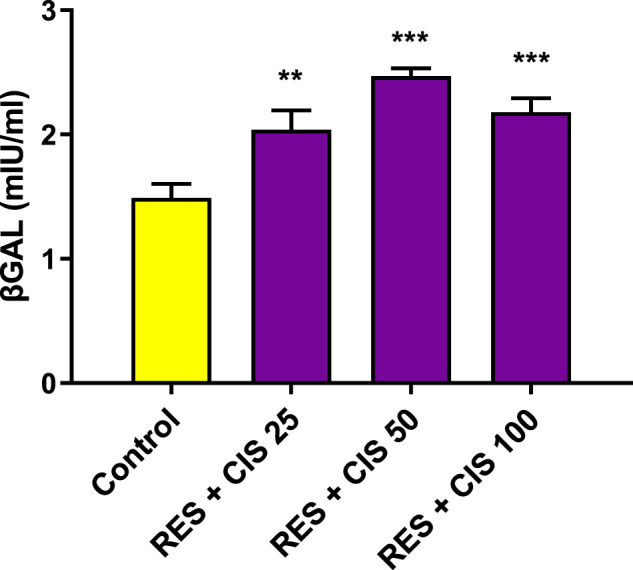
Effect of resveratrol (RES) and different concentrations of cisplatin (CIS) (25, 50 and 100 μg/ml) on the activity of ß-galactosidase (ßgal) enzyme in adenocarcinoma gastric cells (AGS). The results are expressed as mean ± SD performed on *n* = 6, with two independent replicates. *** (*p* < 0.001) and ** (*p* < 0.01) show a significant difference compared to the control group.

The effects of resveratrol and different concentrations of cisplatin (25, 50 and 100 μg/ml on ß-galactosidase activity are shown in Figure 2. Following exposure of AGS cells to cisplatin and resveratrol, there was a significant difference (*p* < 0.001) in ß-galactosidase activity level compared to the control group. The highest activity was observed at concentrations of 50 and 100 μg/ml of cisplatin co-administered with 100 μM of resveratrol.

### 3.4 Effect of resveratrol and different concentrations of cisplatin on the amount of ROS in AGS cells

**FIGURE 3:**
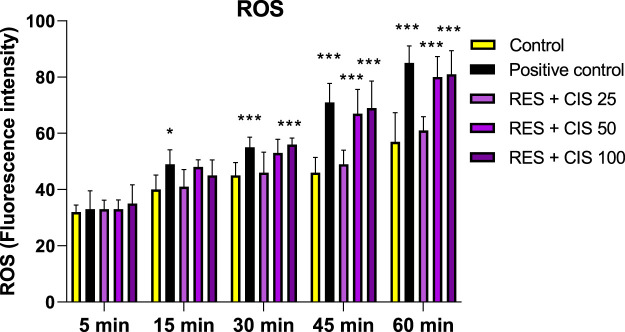
Effect of 100 μM H_2_O_2_ as a positive control, and 100 μM resveratrol (RES) and different concentrations of cisplatin (CIS) (25, 50 and 100 μg/ml) on the amount of reactive oxygen species (ROS). The results are expressed as mean ± SD performed on *n* = 6, with two independent replicates. *** (*p* < 0.001) and ** (*p* < 0.01) show a significant difference compared to the control group.

As shown in Figure 3, resveratrol and all concentrations of cisplatin increased the level of ROS compared to the control group. The significant rise was observed in the groups receiving resveratrol and cisplatin at concentrations of 50 and 100 μg/ml (*p* < 0.001). It should also be noted that the highest amount of ROS was observed after 60 min from the start of the reaction in the groups receiving resveratrol and cisplatin at the concentration of 100 μg/ml.

### 3.5 Effect of resveratrol and different concentrations of cisplatin on the expression of genes

As summarized in Table 1, the expression level of *p53*, *p38*, *p21* and *p16* genes in the groups exposed to resveratrol and cisplatin significantly elevated as the concentration increased compared to the control group. The highest rise in expression of *p53*, *p38* and *p21* genes were seen at the concentration of 100 μg/ml cisplatin (3.25, 3.87, 1.94 times, respectively, with a significancy levels of *p* < 0.001, *p* < 0.05 and *p* < 0.001). The results also demonstrated that the expression levels of pro-inflammatory genes, *TNF-*α, *IL-1*β, *IL-6* and *MMP-2*, were reduced in the groups exposed to both resveratrol and cisplatin. The highest reduction was seen at the concentration of 100 μg/ml cisplatin.

**TABLE 1:** Effect of resveratrol (RES) and different concentrations of cisplatin (CIS) on *p38, p53, p21, p16, TNF-*α*, IL-1*β*, IL-6* and *MMP2* cell migration marker expression level. The β-actin gene has been used as the housekeeping gene.

Experimental groups (relative fold change ±SD) relative to the control group			
RES + CIS in different concentrations (µg/ml)			
Genes	25	50	100
p38	1.28 ± 0.213	1.80 ± 0.514**	1.87 ± 0.563***
p53	1.59 ± 0.563**	3.16 ± 0.661**	3.25 ± 1.176 *
p21	1.38 ± 0.220	1.96 ± 0.465*	3.94 ± 0.759***
p16	1.19 ± 0.220	1.23 ± 0.563**	1.16 ± 0.416

TNF-α	0.71 ± 0.220	1.01 ± 0.294	0.16 ± 0.196***
IL-1β	0.64 ± 0.220	0.97 ± 0.171	0.44 ± 0.049**
IL-6	0.49 ± 0.073**	0.37 ± 0.098***	0.10 ± 0.220**

MMP2	0.02 ± 0.024***	0.11 ± 0.220***	0.15 ± 0.196***

The results are expressed as mean ± SD, performed on *n* = 6, with two independent replicates. *** (*p* < 0.001), ** (*p* < 0.01) and * (*p* < 0.05) show a significant difference with the control group.

### 3.6 Effect of resveratrol and different concentrations of cisplatin on cell cycle distribution in AGS cells

**FIGURE 4:**
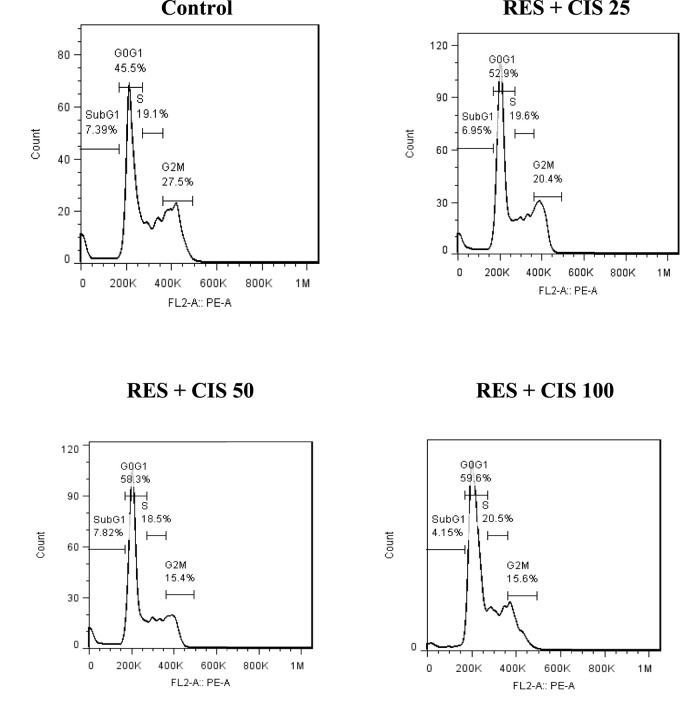
Effect of resveratrol (RES) and different concentrations of cisplatin (CIS) (25, 50 and 100 μg/ml) on cell cycle distribution phases.

As shown in Figure 4, the cell cycle distribution in AGS cancer cells exposed to resveratrol and different concentrations of cisplatin in phases of G0/G1, S and G/M was determined. In the control group, about 45.5% of mouse fetal fibroblasts were in the G1 phase, 19.1% in the S phase, 27.5% in the G2/M phase and 7.39% in the sub-G1 phase. In the cells exposed to resveratrol and cisplatin, elevated cisplatin concentration was associated with a rise in the rate of cell arrest at the G0/G1 stage. The highest rate of cell migration in the G1 phase was observed in cells exposed to 100 μg/ml cisplatin.

### 3.7 Effects of cisplatin and resveratrol on cell migration by scratch in AGS cells

**FIGURE 5:**
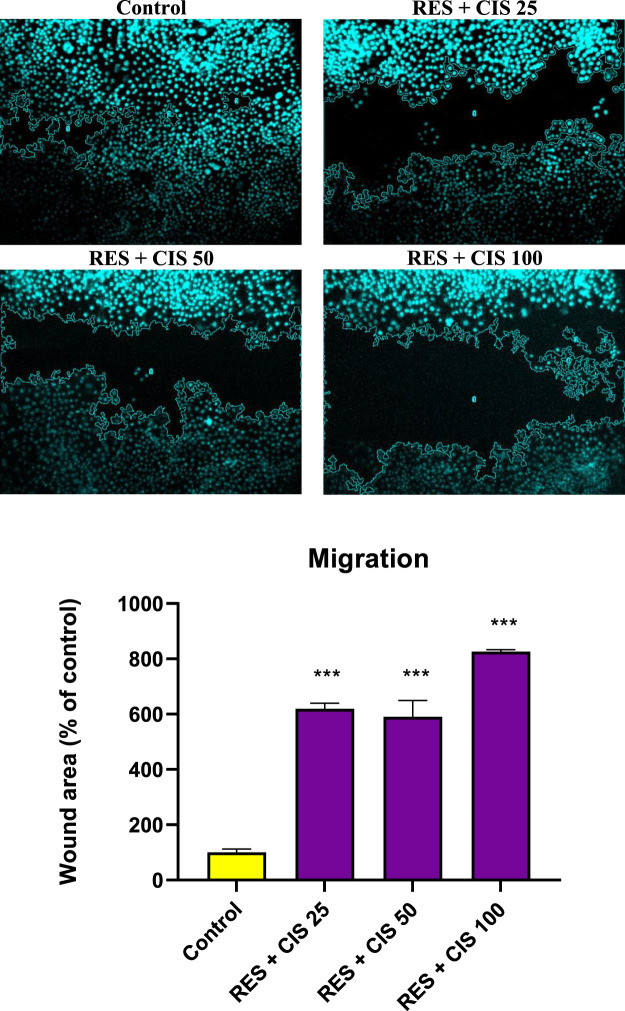
Effect of resveratrol (RES) and different concentrations of cisplatin (CIS) (25, 50 and 100 μg/ml) on the scratch area of adenocarcinoma gastric cells (AGS). Fluorescent microscope images were taken from ×20 magnified cell scratches and analyzed using ImageJ software. The results are expressed as mean ± SD performed on *n* = 6, with two independent replicates. *** shows significant change in cell migration compared to the control group (*p* < 0.001).

The effect of various doses of cisplatin and resveratrol on the pace of cell migration and the change in cell scratch area of human AGS is depicted in Figure 5. In the control group, which did not receive any agents, the cells migrated to the scratch location 48 h after the scratch and covered most of the area. This can be seen in the microscopic image. On the other hand, the rate of cell migration was dramatically slowed down in the groups that were given treatment with resveratrol plus cisplatin. In addition, the scratch level was significantly (*p* < 0.001) higher than the group that served as the control. The group that was given resveratrol and cisplatin at a dose of one hundred micrograms per milliliter exhibited the slowest rate of cell migration.

### 3.8 Effect of resveratrol and cisplatin on telomerase activity in AGS cells

**FIGURE 6:**
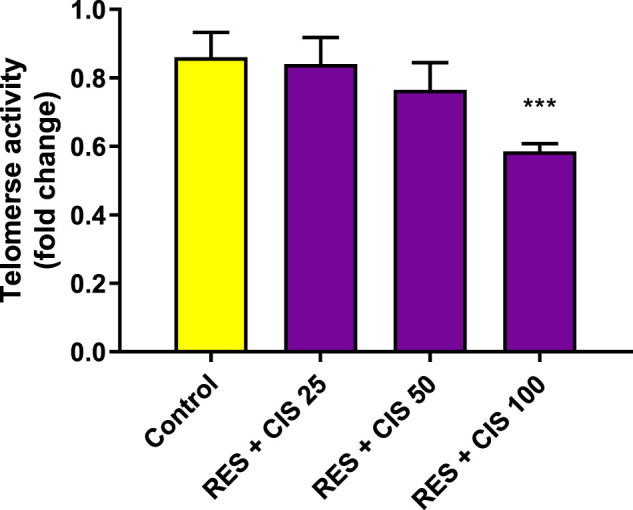
Effect of resveratrol (RES) and different concentrations of cisplatin (CIS) (25, 50 and 100 μg/ml) on telomerase activity in adenocarcinoma gastric cells (AGS). The results are expressed as mean ± SD performed on *n* = 6, with two independent replicates. ** (*p* < 0.01) indicates a significant difference with the control group.

As depicted in Figure 6, the level of telomerase activity in the control group and the groups receiving resveratrol and cisplatin at the concentrations of 25 and 50 μg/ml was not accompanied with a significant difference. The results showed that cells exposed to the concentration of 100 μg/ml of cisplatin had less telomerase activity than the control group (*p* < 0.01).

## 4 Discussion

**FIGURE 7:**
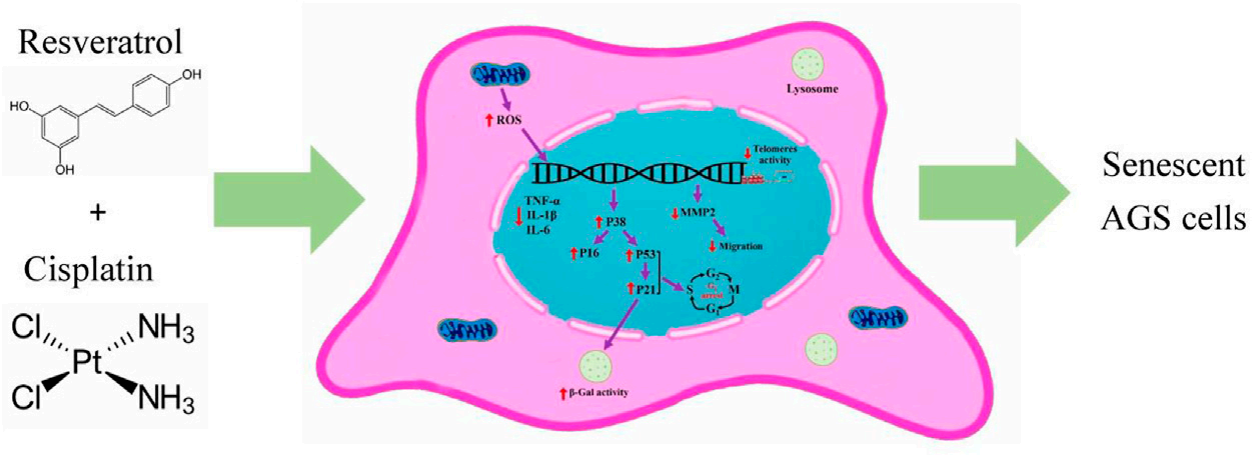
The crucial mechanisms involved in present study. Cisplatin and resveratrol in appropriate dose induced aging in AGS cell line. ↑increased; ↓decreased; ROS, reactive oxygen species; IL-6, interleukin six; IL-1β, Interleukin one beta; TNF-α, tumor necrosis factor alpha; MMP2, matrix metalloproteinase-2.

Within this experiment, the synergistic effect of cisplatin and resveratrol on the induction of cell senescence and metastasis reduction were evaluated using AGS cell line. Some of the crucial mechanisms involved in present study are illustrated in the Figure 7.

Chemotherapy is an efficient therapeutic option for a number of cancers, but its clinical use is constrained by a number of its side effects, including nausea, vomiting, mucositis, and non-specific harm to other tissues (Samadi et al., 2021). The main obstacles to cancer chemotherapy are resistance to chemotherapeutic medicines, having cytotoxic effects on normal cells, and unsatisfactory results. According to a survey, some natural substances can boost the efficiency of conventional chemotherapy medications (Najafi et al., 2020).

Gastric cancer palliative care, including chemoradiation therapy has been associated with non-specific mechanism of action and several adverse drug reactions in most of the cases. More to the point is the substantial incidence and economic burden of the disease imposed on patients and governments. This makes disease management to be even more challenging in resource-limited regions (Li et al., 2022), suggesting a prompt need to introduce cost-effective and available chemotherapy agents.

As one of the main components of ECF and ECX regimens, cisplatin is commonly used in the management of gastric cancer. However, its systemic toxicity and resistant progression is still concerning in the clinical practice. To this end, cisplatin has been combined with boswellic acid (Al-Bahlani et al., 2020), vitamin C (Ghavami and Sardari, 2020) and (Sun et al., 2019) *in-vitro*, showing promising apoptotic and anti-cancer effects. The synergistic effect of resveratrol and cisplatin, in view of cell viability inhibition, G2/M phase arrest and endoplasmic reticulum stress-mediated apoptosis induction was also reported in another study (Ren et al., 2020). In line with this, the current study was carried out to more deeply investigate effects of resveratrol on cytotoxicity, cellular aging, cell migration and inflammation of gastric tumor cells.

There may be therapeutic properties to resveratrol. For tumors like stomach cancer, the chemotherapy combination therapy is adopted as a conventional treatment. Due to their proven remarkable advantages for minimizing chemotherapy side effects on normal cells and their chemosensitizing properties in cancer cells, resveratrol-containing materials are being studied to learn more about the positive elements of utilizing them in combination with chemotherapy (Xiao et al., 2018). Resveratrol has been shown to be safe at high concentrations, despite reports that it has therapeutic benefits in treating cancer at low doses (Salehi et al., 2018; Ashrafizadeh et al., 2021b).

Telomere disruption, oxidative stress, and oncogenic activation are only a few causes of cellular senescence. Senescent cell buildup accelerates the aging process. Senescent cells differ from cycling or resting cells in a number of ways, including an increase in the quantity of cyclin-dependent kinase inhibitors and the emergence of β-galactosidase activity (Momtaz et al., 2019).

Cellular aging is generally recognized as cell cycle arrest that occurs for a variety of reasons. The mechanism of cell cycle arrest due to cellular aging is complex, involving the interactions between telomere shortening, inflammation, and oxidative stress (Kumari and Jat, 2021), though the latter shows biphasic impacts based on the level of ROS. This process is associated with permanent destructions to macromolecules along with biological pathways, eventually inhibiting cell proliferation and resulting in cell senescence. Looking differently, optimal level of ROS activates p16/p21 cellular pathway responsible for cellular arrest in the G1 stage and cell senescence. Further, cell senescence exerts its beneficial effect through suppressing tumorigenesis. Albeit, when accumulated, senescent cells may exacerbate the condition in a negative way. ROS also induce p53/p38, another pathway leading to cell senescence (Poulsen et al., 2014; Ribezzo et al., 2016; Pyo et al., 2020; Haghi-Aminjan et al., 2021).

Numerous researches have investigated the anti-cancer benefits of resveratrol in all stages of cancer initiation, promotion and progression (Schmitt, 2007; Baxter, 2008; You et al., 2019; Pyo et al., 2020).

Although the exact mechanism of action has not been fully defined yet, resveratrol induced cell senescence, cell cycle arrest, and increased anti-oxidant capacity (Rojo et al., 2022) (decreased the invasion) as well as reducing metastasis in former preclinical studies (Pyo et al., 2020). However, its biological effects might vary considering different cell types (Farhadnejad et al., 2019). For instance, both anti-oxidative and pro-oxidative profile has been seen with resveratrol depending on the context (Farhadnejad et al., 2019). Our study supports the pro-oxidant action of resveratrol against AGS cell line. That is, resveratrol and all concentrations of cisplatin increased the level of ROS compared to the control group. Significant effects were reported with cisplatin at concentrations of 50 μg/ml (after 45 and 60 min) and 100 μg/ml (after 30, 45 and 60 min). Considering telomerase activity, cells exposed to resveratrol together with cisplatin at the concentration of 100 μg/ml, had substantially less telomerase activity than the control group, suggesting senescence induction. Additionally, the expression levels of *p53*, *p38*, *p21* and *p16* genes in the groups exposed to resveratrol and cisplatin, particularly at the concentration of 100 μg/ml were significantly elevated compared to the control group. Up-regulation of *p53* protein by resveratrol in AGS cells was also indicative of senescence in another study (Riles et al., 2006).

As expected, co-administration of resveratrol and cisplatin caused cell arrest at the G0/G1 stage in a dose-dependent manner. This is while, resveratrol-cisplatin combination imposed G2/M phase cell arrest (Ren et al., 2020). Last but not least, ß-galactosidase activity, as the most important marker of cellular aging, was significantly elevated in all concentrations of cisplatin-resveratrol treated AGS cell line; like the previous report on glioma cells (Farhadnejad et al., 2019). Besides affecting cellular aging and oxidative stress, resveratrol has been reported to have anti-inflammatory features against cancerous cells (Udenigwe et al., 2008; Haghi-Aminjan et al., 2018). The current study also demonstrated that the expression level of inflammatory genes, *TNF-*α, *IL-1*β, *IL-6* and *MMP-2*, was reduced in the groups exposed to both resveratrol and cisplatin at the highest concentration (100 μg/ml). Inflammation has also been linked to aging, and oxidative stress has been implicated in pro-inflammatory processes. Pro-inflammatory cytokines such *TNF-*α*, IL-1*β, and *IL-6* are down-regulated when inflammation develops in AGS cells. Resveratrol therapy considerably decreased the levels of *TNF-*α*, IL-1*β, and *IL-6* in the current study when compared to the control group. It implies that resveratrol can cause a decrease in AGS inflammation. According to this study, resveratrol’s anti-inflammation properties may have contributed to the therapy’s reduced activation status of AGS.

It is reported MMP-2 is activated by several parameters including ROS (Svineng et al., 2008), osteopontin (Zhang et al., 2011), TGF-*β*2 (Baumann et al., 2009) and TIMP-2 (Itoh et al., 2001; Rosenthal and Matrisian, 2006), among other mechanisms.

The promising advantage of resveratrol on preventing AGS cells invasion and metastasis was stated *in-vitro*, especially at the concentrations of 5–25 µM (Rojo et al., 2022). Almost similarly, the current experiment proved a reduction in cells migration following treatment of AGS cells with cisplatin-resveratrol combination, at all concentrations. Considering all these, our study suggested that the right dose of resveratrol-cisplatin combination could trigger cell senescence in AGS cell line. Moreover, the date of present study indicated resveratrol reduced the effective dose of cisplatin to induce senescence on AGS cells.

## 5 Conclusion

According to the current experiment, it can be concluded that resveratrol and cisplatin synergistically impeded the proliferation AGS cell line, reduced cells invasion, and telomerase activity along with the induction of apoptosis, ß-galactosidase activity and cell cycle arrest at the G0/G1 stage. These effects are mediated through targeting P16/P21 and P38/P53 pathways as well as alternating the level of ROS and pro-inflammatory cytokines. These promising results will, indeed, pave the way for additional preclinical and clinical studies on combination of polyphenols, particularly, resveratrol, with other chemotherapy agents. Further investigations might result in the emergence of cost-effective, available, and accessible treatment regimens with the specific targeted action and the least possible adverse drug reactions.

## Data Availability

The original contributions presented in the study are included in the article/supplementary material, further inquiries can be directed to the first authors; MR (mahban.rahimifard@gmail.com), MB (baeeri.maryam@gmail.com).
